# Framing diagnostic error: an epidemiological perspective

**DOI:** 10.3389/fpubh.2024.1479750

**Published:** 2024-12-09

**Authors:** Montana Kekaimalu Hunter, Chithra Singareddy, Kenneth A. Mundt

**Affiliations:** ^1^Stantec ChemRisk, Boston, MA, United States; ^2^Harvard T H. Chan School of Public Health, Department of Epidemiology, Boston, MA, United States; ^3^Frank H. Netter MD School of Medicine at Quinnipiac University, North Haven, CT, United States; ^4^Department of Epidemiology, Boston University School of Public Health, Boston, MA, United States; ^5^Department of Biostatistics and Epidemiology, University of Massachusetts, Amherst, MA, United States; ^6^Society to Improve Diagnosis in Medicine, Alpharetta, GA, United States

**Keywords:** epidemiology, medical misdiagnosis, health equity, diagnostic error, risk factors, societal implications

## Abstract

Diagnostic errors burden the United States healthcare system. Depending on how they are defined, between 40,000 and 4 million cases occur annually. Despite this striking statistic, and the potential benefits epidemiological approaches offer in identifying risk factors for sub-optimal diagnoses, diagnostic error remains an underprioritized epidemiolocal research topic. Magnifying the challenge are the array of forms and definitions of diagnostic errors, and limited sources of data documenting their occurrence. In this narrative review, we outline a framework for improving epidemiological applications in understanding risk factors for diagnostic error. This includes explicitly defining diagnostic error, specifying the hypothesis and research questions, consideration of systemic including social and economic factors, as well as the time-dependency of diagnosis relative to disease progression. Additional considerations for future epidemiological research on diagnostic errors include establishing standardized research databases, as well as identifying potential important sources of study bias.

## Introduction

Diagnostic errors remain a grossly under-assessed patient safety and quality of care threat (1–3). Annual estimates of diagnostic error vary widely, from 40,000 to 4 million cases nationwide. Over half are related to cardiovascular diseases, infections, and cancers, and over 6% of these result in serious harm to patients (4). Newman-Toker et al. estimated that 5.7% of all emergency department visits in the United States involve some diagnostic error, affecting 7 million patients annually. Furthermore, 0.3% of all patients suffer from related preventable and serious harm, including disability or death (5). Notably, the published literature tends to focus on medication errors, surgical complications, or healthcare-acquired infections rather than medical diagnostic error (6).

Challenges in evaluating the etiological underlying causes of diagnostic error stem from methodological, logistical and social considerations. Factors such as the emergence of new diseases, evolution of diagnostic capabilities, and advancement of clinical medicine and research complicate defining diagnostic error (7). Initial disease diagnosis may be more subjective due to a lack of confirmatory testing, and greater reliance on provider experience and medical cognition. Diagnostic errors can be obscured when multiple clinicians concur with a given diagnosis and sometimes only revealed retrospectively or after a complaint is raised (3, 8). Moreover, variations in clinical thresholds for testing (including resource limitations) undermine standardization. Consequently, definitions of diagnostic errors may be subject to the perspectives and responsibilities of the defining stakeholder; clinicians, patients, and researchers likely have different measures and purposes for defining events (or omissions) as “errors.”

Epidemiological analysis can reveal patterns in rates, types, risk factors and root causes. A standard framework using epidemiological tools can be applied across diseases and clinical presentations and settings however, all such research will require valid definitions of the diagnostic error outcomes. Ultimately, identifying the factors that result in diagnostic errors subsequently can inform strategic mitigation approaches and improve patient safety.

In this narrative review, we present several dimensions for improving epidemiological research on diagnostic error. Key considerations include accuracy of diagnosis, the relative timing of diagnosis, data sources and their accuracy and completeness, social determinants of diagnostic options, and potential sources of bias including those associated with the patient (e.g., obesity, race, affluence, etc.), the provider (e.g., training and experience, level of interest and commitment, etc.) and the clinical setting (e.g., academic vs. commercial institutions, business and revenue models, insurance structures, etc.). Our primary objective is to illuminate the complex landscape of diagnostic error research, highlighting the nuanced challenges of using epidemiologic methods to analyze the roots of diagnostic error.

## Defining diagnostic error

The Institute of Medicine (IOM) defines diagnostic error as “*the failure to (a) establish an accurate and timely explanation of the patient’s health problem(s) or (b) communicate that explanation to the patient*” (9, 10). However, this is one of several definitions (see Table 1). Schiff et al. (2009) offers greater scope, defining diagnostic error as “any mistake or failure in the diagnostic process leading to a misdiagnosis, a missed diagnosis, or a delayed diagnosis (11).” This broader definition highlights diverse types of errors, such as wrong, overlooked and delayed diagnosis, and considers both the method and timing of diagnosis (11) (Table 1).

**Table 1:** Diagnostic error: select definitions (1, 9–11, 19, 32–34).

Term	Definition	Citation
Diagnostic error	“The failure to (a) establish an accurate and timely explanation of the patient’s health problem(s) or (b) communicate that explanation to the patient”	IOM
Misdiagnosis-related harms	“Harms resulting from the delay or failure to treat a condition actually present (false-negative diagnosis) or from treatment provided for a condition not actually present (false-positive diagnosis)”	Newman-Toker et al. (2009) and (2014)
Diagnostic error	“Diagnosis that was unintentionally delayed (sufficient information was available earlier), wrong (another diagnosis was made before the correct one), or missed (no diagnosis was ever made), as judged from the eventual appreciation of more definitive information”	Graber et al. (2005)
Diagnostic error	“Any mistake or failure in the diagnostic process leading to a misdiagnosis, a missed diagnosis, or a delayed diagnosis”	Schiff et al. (2009)
Diagnostic error	“Implies that something different could have been done to make the correct diagnosis earlier. Evidence of omission (failure to do the right thing) or commission (doing something wrong) exists at the particular point in time at which the ‘error’ occurred”	Shojania et al. (2003)
Diagnostic error	“1. Case analysis reveals evidence of a missed opportunity to make a correct or timely diagnosis. The concept of a missed opportunity implies that something different could have been done to make the correct diagnosis earlier. The missed opportunity may result from cognitive and/or system factors or may be attributable to more blatant factors, such as lapses in accountability or clear evidence of liability or negligence. 2. Missed opportunity is framed within the context of an “evolving” diagnostic process. The determination of error depends on the temporal or sequential context of events. Evidence of omission (failure to do the right thing) or commission (doing something wrong) exists at the particular point in time at which the “error” occurred. 3. The opportunity could be missed by the provider, care team, system, and/or patient. A preventable error or delay in diagnosis may occur because of factors outside the clinician’s immediate control or when a clinician’s performance is not contributory. This criterion suggests a system-centric versus physician-centric approach to diagnostic error.”	Singh et al. (2005)
Diagnostic error	Undesirable diagnostic events as specific, measurable, and actionable clinical situations likely to denote the presence of diagnostic error.	Olsen et al. (2018)

The diagnostic process is complex, evolving over time and involving multiple stakeholders, which can complicate finding the sources of error (12). This complexity introduces an intriguing challenge: addressing diagnostic error as a time-dependent phenomenon. Therefore, “error” can be defined in two ways: deviation from “the truth” or, departure from what reasonably (or expertly) could be achieved based on the available information at specific stages of the disease. For instance, at the earliest stages of a disease process, diagnostic ability is limited by the available indications, but as the disease progresses, additional test results, signs and symptoms may provide increasingly clear diagnostic clues.

A standardized definition of diagnostic error is important for both comparability across studies and validity and reproducibility of individual study findings.

## Partitioning diagnostic error

Considering the range of possible sources of diagnostic error may help refine the hypotheses and research questions in designing epidemiological studies of diagnostic error. Graber et al. (2005) further classified diagnostic errors into three categories: no-fault errors, system-related errors, and cognitive errors (8).

No-fault errors, as described by Kassirer and Kopelman, are influenced by factors that are outside of clinician or system control, such as atypical disease presentation and patient-related factors. These include inaccurate or incomplete information that leads clinicians down an incorrect diagnostic reasoning path (13). Factors related to the patient, healthcare team, or care environment may also compromise diagnostic acuity.

System-related errors are those caused by technical failures, equipment problems, organizational challenges, lack of communication among healthcare teams, or inefficient processes at the system-level (1, 7). The harms of missed diagnoses or misdiagnoses from system-related errors may be more pronounced among marginalized populations, such as racial minorities or the older adults, due to implicit biases or assumptions that impede clinical reasoning.

Cognitive errors are clinician-based mistakes made despite the clinician possessing the ability to make the correct diagnosis, possibly due to subconscious biases (14). They are rooted in implicit biases, confirmation biases, inadequate training or knowledge, poor critical thinking or competency, or failure to fully investigate and gather information (1, 13). The bias of anchoring to premature or initial findings or recent and memorable interactions can create a confirmation bias favoring the evidence of a working hypothesis the clinician may have already had. Additionally, fatigue, stress, or burnout may reduce diagnostic capacity (6).

Diagnostic error likely occurs as the product of multiple partitioning factors occurring simultaneously, including individual perspectives, patient and disease complexities, and systematic practices. Epidemiological study designs and analytical approaches will need to anticipate and accommodate not only easily measured medical and patient characteristics, but also more abstract behavioral, cultural and likely interdependent (i.e., non-independent) variables and factors.

## Epidemiological framework

In traditional etiological epidemiological research, the goal often is to identify factors associated with a disease outcome that, if removed, would have prevented that outcome from occurring. Similarly, one might frame this in terms of how the diagnostic outcome would have differed had one or more modifiable “risk” factors not operated or been present. However, diagnostic errors present an additional challenge: they may never be detected or may be impossible to measure directly. This makes it particularly difficult to establish a reliable referent or “gold standard”—also counterfactual—against which the observed diagnostic is compared. Where a disease or disease process is evolving, the expectation that a firm and accurate diagnosis is uniformly possible may be unrealistic. Furthermore, diagnosis is fluid, and often is reformulated, revised or refined over time; therefore ultimately, the diagnostic error *per se* may not solely represent the presumably adverse outcome, but rather, the consequences (possibly in terms of actions or inaction) that result from it. This expands the scope of “risk” factors for diagnostic error to include information that might not be available or obtainable in a given situation (for any number of reasons): in theory, had it been available a more favorable outcome might have been possible.

Therefore, rather than striving for a standardized definition of diagnostic error, we recommend critically thinking about how timing and accuracy of a diagnosis impact its accuracy and validity, and further, what the consequences would be of treatment decisions that rely on the current diagnosis; the possible associated partitioning errors; the validity of available (or derived) data sources and their utility for identifying and characterizing errors; potential sources of biases; and key sociodemographic aspects that might play a role. This provides a general critical framework for studying diagnostic error with epidemiological methods, but with special consideration of the ‘non-traditional’ study aspects, as well as the flexibility required to address complex systems in which diagnoses are generated, and the diverse responses they may impact. Well known in epidemiology is the potential harm associated with early screening and detection of diseases for which early treatment is not measurably better, especially if psychological stress is induced. Timing and accuracy could then be considered potential confounders or effect modifiers, respectively.

Under the suggested framework, patient-provider interactions that delay the communication of a diagnosis to the patient should be analyzed as if the timing of diagnosis is when the patient received the diagnosis. In situations where the initial diagnosis is revised or improved, additional time to accurate diagnosis accrues. The timing of communication of diagnosis to the patient is important because it marks the beginning of a patient’s ability to self-manage their condition, which can affect their ultimate health outcome.

As in all epidemiological applications, study design will vary based on the proposed research questions and objectives. If the primary aim is to understand the overall impact of diagnostic error on patient outcomes, focusing on accuracy might be more appropriate, irrespective of timing, as well as how the diagnosis influenced treatment or other care. Alternatively, if evaluating the timing of diagnostic error is the objective, a baseline timeline or scale will need to be derived (as a referent), such that diagnoses rendered at various times over the natural history of a disease process appropriately will be compared with what might be attainable at each stage. Consequently, the point at which a patient seeks medical care may affect the diagnostic process and the likelihood of diagnostic error. Seeking care may be delayed until symptoms are pronounced, which might make the diagnosis more straightforward. On the other hand, more advanced disease may be accompanied by comorbidities, or reflect atypical signs and symptoms, and in general, over time, underlying risk factors or even causal factors will become distanced from their effects, possibly obscuring the ability to observe a relationship.

**Figure 1:**
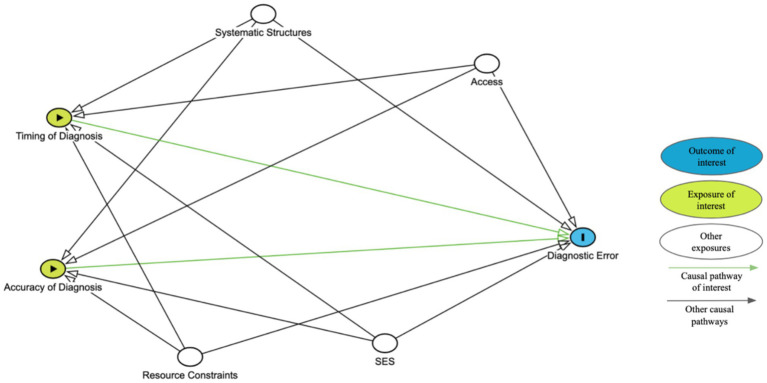
Simplified DAG template to guide analyses.

Figure 1 presents a simplified directed acyclic graph, or DAG, to illustrate the hypothetical causal relationships between timing or accuracy and diagnostic error. Other nodes may need to be added based on the population of interest. This diagram should be used in conjunction with a clearly defined study design, appropriate statistical analysis, and explicitly stated assumptions to determine the strength of association between timing or accuracy of diagnosis and diagnostic error.

We acknowledge that the relationship between timing and diagnostic acuity is complex making it difficult to separate the effects. Advanced epidemiological analytical techniques such as path analysis may help researchers clarify the direct and indirect effects of timing and accuracy on diagnostic errors and patient outcomes. However, the decision should be guided by the specific research questions, available data, and understanding of these and other possibly intercorrelated factors. Maintaining transparency in the study methods allows for interpretable, replicable, and ideally, more applicable and improved results.

## Other framework considerations

### Data sources

Studying diagnostic error from an epidemiological perspective necessitates careful evaluation of available data sources, each with their possible strengths and limitations. These sources could include electronic health records, laboratory and imaging tests, autopsy reports, and health insurance or malpractice claims data—or more subjective sources such as patient care feedback or research-initiated patient surveys. By understanding the specific contexts in which misdiagnoses commonly occur, it is hoped that healthcare systems will be better equipped to devise targeted strategies to prevent these errors (15, 16).

The largest data sources for examining diagnostic errors are administrative databases such as EPIC or Cerner (17). These repositories of medical information are collected and maintained by hospitals, clinics, and insurance companies that include information such as medical claims, records of service, prescriptions, procedures, and diagnoses of patient’s medical encounter (14). Retrospective analysis of these data allows researchers and clinicians to assess geographic, demographic, and temporal patterns of diagnostic error. It also facilitates the exploration of system-level factors, such as the relationship between medical institutions’ staffing or patient volume and the rate of diagnostic error. Using large administrative databases also allows estimation of population-level diagnostic error rates across medical conditions, the investigation of sociodemographic disparities in diagnostic error, and the study of diagnostic error’s impact on care progression. However, there are challenges to the retrospective use of administrative databases. These include the time-consuming nature of review, as sometimes manual review is required for paper-based documentation, and the potential for incomplete or inaccurate information, which may affect the sensitivity and specificity of the method. These limitations can restrict the epidemiological methods available for data analysis (17).

Autopsy reports, pathology findings, clinical health summaries, and interpretative summaries, often reveal significant diagnostic errors (18). It was estimated that historically, as much as 25% of autopsies uncover some form of major diagnostic error, and 10% revealed errors directly contributing to the death; encouragingly, the rate of diagnostic errors discovered at autopsy has declined (19). Schwanda-Burger et al., assessed diagnostic errors in a teaching hospital and reported a 23% decrease in errors found in autopsies over a 30-year period, attributing this decline to improved technology, medical records, and diagnostic tools (20).

Much like large administrative databases, medical malpractice claims data can provide insight into the geographic, demographic, and temporal context of misdiagnoses. These data also can reveal which conditions are most frequently misdiagnosed, although they likely reflect selection biases favoring documented examples where serious harm resulted. For instance, vascular events, infections, and cancers constituted 72% of all emergency department diagnostic errors resulting in serious harm (5). Patients with more serious conditions or harmful outcomes are more likely to file malpractice claims, whereas those who did not experience serious harm are less likely to file claims (9). However, medical malpractice claims data may exclude groups who lack the knowledge or financial ability to file malpractice claims. Such claims also might be falsely brought, motivated by potential monetary gains. There may also be gaps related to the misdiagnosed condition and the diagnostic error’s outcome.

When designing studies and interpreting results, epidemiologists carefully should consider the strengths and weaknesses of available data resources. Understanding the completeness, inherent biases, uncertainties and ultimately the validity of selected data sources can lead to the improvement of study methods and the interpretation of findings. By being transparent, epidemiologists can contribute to a better understanding not only of factors associated with diagnostic error, but to recommendations on improving the approaches and study designs used to evaluate diagnostic errors and their risk factors and possibly their root causes.

### Social determinants

Epidemiological research to identify risk factors for diagnostic error additionally requires definition and measurement of a range of indicators and phenomena including individual patient constitutional characteristics (e.g., age, sex, race/ethnicity, educational level, economic access, etc.), location characteristics (e.g., urban, rural, state/region, country); clinical factors (e.g., type of facility, provider attributes including occupation, specialty, education, etc.); access and other socioeconomic factors; and diseases or conditions of interest that may not be well-known or defined. Though there are several social determinants to consider, we note that access to care, resource constraints, and systemic structures can significantly impact timing and accuracy of diagnosis and diagnostic error.

### Access

Access to medical care impacts patients’ ability to be diagnosed and treated, and likely the quality of these as well. The ability to access and obtain medical care from competent and resourced local providers is not universal, even in highly advanced societies. Medical costs, insurance, transportation, mobility, desire and fear are just a few of the factors that may influence an individual’s access to medical attention that could benefit their present and future well-being (21). Greater access to care allows an individual to see a provider sooner and possibly to see multiple providers, theoretically improving the probability of obtaining accurate diagnoses and treatments, including seeking second opinions. Thus, access to medical care should be measured and explored as potential risk factors or effect modifiers in epidemiological studies of diagnostic error.

### Resource constraints

Medical care quality may be constrained by institutional resource availability and management. High-and low-resource settings, regions, or systems where healthcare resources are abundant or limited, respectively, are accessed by an institution’s financial capacity, technology, workforce ability, care quality, and research program. A region or institution with access to more testing, information, and colleagues to discuss complex patient presentations, allows (at least conceptually) for more accurate and timely diagnoses (22). When the resource setting has more diverse and culturally competent healthcare workers, cultural and language barriers can be more appropriately addressed. This allows patients to feel more capable of effectively participating in their care because the diagnostician is more likely to listen to and understand the patient’s symptoms, personal situation, sociodemographic background, cultural perspectives, and beliefs that inform the case and associated research, which is key for better diagnostic care (22). Nevertheless, for more common and less serious medical conditions, no more than basic clinical resources and personnel may be required for accurate diagnoses and appropriate treatment; therefore, the level of consideration of resource constraints also will depend on the research questions epidemiologically being addressed.

### Systemic structures

Systemic structures of healthcare involve the interplay of people, resources, processes and institutions, including critical communication pathways among these and ultimately with the patient (23). In epidemiology, these systemic structures are often considered by controlling for confounders such as insurance type or hospital location, tangible entities that are measurable. However, systemic structures are often intangible like power dynamics and agendas of larger institutions that are complex and challenging to define (24). These systemic structures influence the way diagnostic processes are designed, structured, and evaluated. Systemic structures influence the allocation of resources, funding, and training which can impact the accuracy and timing of diagnoses (25). When establishing a study design, it is crucial to acknowledge and account for social determinants (e.g., socioeconomic status, race) that act as proxies for systemic structures. Explicitly identifying how the social determinant(s) might influence diagnostic accuracy or error can help to elucidate these complicated relationships and reveal that certain factors require more complex adjustment.

### Potential study biases

Bias in epidemiological research refers to methodological or systematic errors that lead to a distortion in the estimated numerical study result, most often a form of relative risk. Biases can lead to errors that range from inconsequential to profound, i.e., where the result misleadingly indicates a risk where one might not exist—or vice versa. Therefore, the design, planning and execution of an epidemiological study provides opportunities to anticipate and reduce the occurrence—or the impact of—specific potential sources of bias. Accordingly, potential sources of bias in evaluating associations between risk factors and the diagnostic error outcomes can be anticipated, identified and mitigated to increase study validity. For example, as discussed under data sources, legal claims databases likely represent a biased subset of medical misdiagnoses, i.e., those more likely to have been well documented, those resulting in serious harm, those occurring among litigious groups and those with the legal resources to pursue their cases. It is likely that this group is select and is not representative of the universe of comparable diagnostic errors.

Medical malpractice is a major concern of healthcare practitioners and has been associated with “defensive medicine” including excessive laboratory testing and increased referrals (16). Over-testing and (over)diagnosis can make identifying and assessing the potential role of risk, confounding and effect modifying (i.e., interdependent) factors challenging. Additionally, clinicians may be more cautious when diagnosing patients with certain characteristics or risk factors, introducing diagnostic bias. Ensuring there are non-punitive reporting systems in place to create and promote safety also would reduce bias. A highlight of this system is maintaining elements of anonymity and confidentiality that can help reduce fear of individual provider blame and encourage more accurate reporting of diagnostic errors. A shift of focus to system-level factors, rather than individual-provider-level factors, may also allow for identification of broader issues that affect diagnostic accuracy.

Nevertheless, The World Health Organization (WHO) identified the training of healthcare providers as a contributor to diagnostic error (26). WHO emphasizes that suboptimal training, specifically lack of training for clinical reasoning and deficient certification and licensure training, contributes to diagnostic error and suggests that clinical training such as embedding decision support tools to assist with differential diagnoses could improve reliable diagnosis. Additionally, training in clinical reasoning, patient safety, critical thinking, and cognitive heuristics may also improve diagnostic accuracy (22). It should be highlighted that no-fault errors such as hours worked, fatigue, management style, and compensation also can impact clinician errors (27); however, it is unclear how these factors would introduce bias into epidemiological studies and whether they might be viewed as independent risk factors or effect modifiers.

Previously discussed as a partitioning effect, cognitive biases further can be categorized as availability heuristic, anchoring heuristic, framing effects, and blind obedience, to name a few (28). Availability heuristic results in the diagnosis of a patient based on provider experience with past cases regardless of the patient’s current presentation. In a randomized control trial of 46 resident physicians, authors found that physicians who were preemptively presented information about dengue fever were more likely to misdiagnose than physicians that had no previous information presented to them (29).

Anchoring heuristic, the reliance on initial diagnostic impressions, despite subsequent information to the contrary, can stem from overconfidence, lower tolerance to risk, and information availability, which is associated with increased diagnostic error. Healthcare workers can unconsciously note data or impressions that “fit” a given diagnosis, but other clues might be discounted (30).

The framing effect is a diagnostic decision due to subtle cues and collateral information. For example, a patient with a history of opioid use and abdominal pain might be treated for opioid withdrawal when they really had a perforation of the bowels (30).

Finally, blind obedience, i.e., placing undue reliance on a test of opinion of “expert” (31). In a behavioral study of paramedic and respiratory therapy students, it was intentionally necessary for students to challenge authority to prevent patient harm. Authors reported that displacement of responsibility was most influential for some participants in not challenging the authority figure’s decision (30). Cognitive biases are common and can significantly influence the diagnostic process leading to patient harm and skew epidemiological data by acting as confounding factors, impacting generalizability, and challenging reproducibility of the study. To minimize the impact of cognitive biases in epidemiological studies, researchers should identify the potential biases in their study design and analysis and understand the role that they may play, addressing the bias accordingly. Alternatively, stratification or more complex analytical parameters such as interaction terms may be needed.

## Discussion

In traditional epidemiology, we often design studies that primarily consider one (or very few) exposure(s) or one (or very few) outcome(s)—often classified binarily, e.g., hepatocellular cancer (yes/no) and hepatitis A/B infections, and several potential confounding factors (e.g., alcohol consumption, cigarette smoking, etc.). Once the specific outcome, risk factor(s) and potential confounding factors are identified, they can be measured and the relationships among them explored and summarized. In diagnostic epidemiology, the definition of the outcome needs to be comparably specific; however, as we discussed above, the definition of diagnostic errors likely will need to consider time-and context-specific aspects—in other words, the diagnostic error may not simply be described accurately in absolute or binary terms of “yes/no.” Additionally, the risk factors and underlying causes of diagnostic error (in general, and also likely for very specific sub-types) will not be measurable chemical or biological agents, but rather multiple abstract and intercorrelated factors including behavioral, social and systemic influences It will be crucial to define these objectively and measure them accurately in diagnostic epidemiological studies.

The timing of diagnosis is especially important as it significantly influences the diagnostic process and most importantly, downstream clinical actions and outcomes. A delayed diagnosis may reduce the risk of an early incorrect one, but also can allow the condition to worsen, leading to complications or atypical presentations that obscure the ultimate diagnosis. Researchers must carefully define what constitutes a “late” diagnosis for their study context. Conversely, accuracy reflects how well the provider’s diagnosis matches the patient’s true underlying condition given the available information at that time. This likely will be challenging to ascertain retrospectively, and therefore might require real-time assessment. Both timing and accuracy themselves may be influenced by several identifiable and measurable factors that impact the ultimate diagnosis. Furthermore, the likely contributors to and determinants of diagnostic errors may act independently—in which case they in theory can be measured and statistically controlled—or effect modifiers, which requires more complex statistical treatment.

Despite the inherent challenges, employing epidemiological principles and methods provides a path forward for improved understanding of diagnostic error from a group (vs. individual patient) level, with promising applications to identifying risk factors and improving patient safety. Ultimately, clearly articulating the research question, defining the specific “diagnostic errors” being evaluated, and leveraging appropriate statistical analytical methods will generate more reproducible research that provides insight into the causes of and factors associated with diagnostic error. The epidemiological approach and methods offer a powerful framework for improving diagnostic science and ultimately healthcare quality.

## Data Availability

The original contributions presented in the study are included in the article, further inquiries can be directed to the corresponding author.

## References

[1] Zwaan L Singh H. The challenges in defining and measuring diagnostic error. Diagnosi. (2015) 2:97–103. doi: 10.1515/dx-2014-0069, PMID: 26955512 PMC4779119

[2] Giardina TD Hunte H Hill MA Layla Heimlich S Singh H. Defining Diagnostic Error: A Scoping Review to Assess the Impact of the National Academies’ Report Improving Diagnosis in Health Care. (2022). 10.1097/PTS.0000000000000999 PMC9698189 35405723

[3] Singh H Schiff GD Graber ML Onakpoya I Thompson MJ. The global burden of diagnostic errors in primary care. BMJ Qual Saf. (2017) 26:484–94. doi: 10.1136/bmjqs-2016-005401, PMID: 27530239 PMC5502242

[4] Newman-Toker DE Schaffer AC Yu-Moe CW Nassery N Saber Tehrani AS Clemens GD. Serious misdiagnosis-related harms in malpractice claims: the “big three” – vascular events, infections, and cancers. Diagnosi. (2019) 6:227–40. doi: 10.1515/dx-2019-0019, PMID: 31535832

[5] Newman-Toker DE Peterson SM Badihian S Hassoon A Nassery N Parizadeh D. Diagnostic Errors in the Emergency Department: A Systematic Review (2022). 36574484

[6] Rodziewicz TL Houseman B Hipskind JE. Medical Error Reduction and Prevention. (2024). 29763131

[7] Graber ML. The incidence of diagnostic error in medicine. BMJ Qual. (2013) 22:ii21–7. doi: 10.1136/bmjqs PMC3786666 23771902

[8] Graber ML Franklin N Gordon R. Diagnostic Error in Internal Medicine. Arch Intern Med. (2005) 165:1493. doi: 10.1001/archinte.165.13.1493 16009864

[9] Balogh EP Miller BT Ball JR. Improving diagnosis in health care. Washington (DC): National Academies Press (2016). 1–472. 26803862

[10] Singh H Meyer AND Thomas EJ. The frequency of diagnostic errors in outpatient care: estimations from three large observational studies involving US adult populations. BMJ Qual Saf. (2014) 23:727–31. doi: 10.1136/bmjqs-2013-002627, PMID: 24742777 PMC4145460

[11] Schiff GD. Diagnostic Error in Medicine. Arch Intern Med. (2009) 169:1881–7. doi: 10.1001/archinternmed.2009.333, PMID: 19901140

[12] Marshall TL Rinke ML Olson APJ Brady PW. Diagnostic error in pediatrics: a narrative review. Pediatrics. (2022) 149:e2020045948D. doi: 10.1542/peds.2020-045948D 35230434

[13] Kassirer JP Kopelman RI. Cognitive errors in diagnosis: instantiation, classification, and consequences. Am J Med. (1989) 86:433–41. doi: 10.1016/0002-9343(89)90342-2, PMID: 2648823

[14] Stiegler M Goldhaber-Fiebert S. Understanding and preventing cognitive errors in healthcare. MedEdPORTAL. (2015) 11. doi: 10.15766/mep_2374-8265.10000

[15] Gupta A Snyder A Kachalia A Flanders S Saint S Chopra V. Malpractice claims related to diagnostic errors in the hospital. BMJ Qual Saf. (2018) 27:53–60. doi: 10.1136/bmjqs-2017-006774, PMID: 28794243

[16] Ostrovsky D Novack V Smulowitz PB Burke RC Landon BE Isbell LM. Perspectives of emergency clinicians about medical errors resulting in patient harm or malpractice litigation. JAMA Netw Open. (2022) 5:e2241461. doi: 10.1001/jamanetworkopen.2022.41461, PMID: 36355376 PMC9650607

[17] Chishtie J Sapiro N Wiebe N Rabatach L Lorenzetti D Leung AA. Use of Epic electronic health record system for health care research: scoping review. J Med Internet Res. (2023) 25:e51003. doi: 10.2196/51003, PMID: 38100185 PMC10757236

[18] Hutchins GM Berman JJ William Moore G Hanzlick R. Strategies for laboratory and patient management practice guidelines for autopsy pathology autopsy reporting. Arch Pathol Lab Med. (1999) 123, 1085–1092 p. 10539932 10.5858/1999-123-1085-PGFAP

[19] Shojania KG Burton EC McDonald KM Goldman L. Changes in rates of autopsy-detected diagnostic errors over time. JAMA. (2003) 289:2849–56. doi: 10.1001/jama.289.21.2849, PMID: 12783916

[20] Schwanda-Burger S Moch H Muntwyler J Salomon F. Diagnostic errors in the new millennium: a follow-up autopsy study. Mod Pathol. (2012) 25:777–83. doi: 10.1038/modpathol.2011.199, PMID: 22362052

[21] Karpman M Zuckerman S Morriss S. Health care access and affordability among US adults aged 18 to 64 years with self-reported post–COVID-19 condition. JAMA Netw Open. (2023) 6:e237455. doi: 10.1001/jamanetworkopen.2023.7455, PMID: 37036705 PMC10087049

[22] van Zyl C Badenhorst M Hanekom S Heine M. Unravelling ‘low-resource settings’: a systematic scoping review with qualitative content analysis. BMJ Glob Health. (2021) 6:e005190. doi: 10.1136/bmjgh-2021-005190, PMID: 34083239 PMC8183220

[23] Clarkson J Dean J Ward J Komashie A Bashford T. A systems approach to healthcare: from thinking to-practice. Futur Healthc J. (2018) 5:151–5. doi: 10.7861/futurehosp.5-3-151, PMID: 31098557 PMC6502599

[24] Kearns E Khurshid Z Anjara S De Brún A Rowan B McAuliffe E. P92 power dynamics in healthcare teams – a barrier to team effectiveness and patient safety: a systematic review. BJS Open. (2021) 5:891–903. doi: 10.1093/bjsopen/zrab032.091

[25] Singh H Onakpoya I Thompson MJ Graber ML Schiff G. Diagnostic errors. (2017) 28 p. 10.1136/bmjqs-2016-005401 PMC5502242 27530239

[26] Singh H Onakpoya I Thompson MJ Graber ML Schiff G World Health Organization, World Health Organization. Diagnostic errors:technical series on safer primary care. (2016).

[27] ALQahtani DA Rotgans JI Ahmed NE Alalwan IA Magzoub MEM. The influence of time pressure and case complexity on physicians′ diagnostic performance. Health Prof Educ. (2016) 2:99–105. doi: 10.1016/J.HPE.2016.01.006

[28] Guidry MM Drennan RH Weise J Hamm LL Hamm LL. Serum Sepsis, not sickness. Am J Med Sci. (2011) 341:88–91. doi: 10.1097/MAJ.0b013e3182083f14 21273840

[29] Li P Cheng ZY Lin LG. Availability Bias causes misdiagnoses by physicians: direct evidence from a randomized controlled trial. Intern Med. (2020) 59:3141–6. doi: 10.2169/internalmedicine.4664-20, PMID: 32788532 PMC7807127

[30] Violato E Witschen B Violato E King S. A behavioural study of obedience in health professional students. Adv Health Sci Educ. (2022) 27:293–321. doi: 10.1007/s10459-021-10085-4, PMID: 34807358 PMC9117351

[31] Ogdie AR Reilly JB Pang WG Keddem S Barg FK Von Feldt JM. Seen through their eyes: residents’ reflections on the cognitive and contextual components of diagnostic errors in medicine. Acad Med. (2012) 87:1361–7. doi: 10.1097/ACM.0b013e31826742c9, PMID: 22914511 PMC3703642

[32] Newman-Toker DE. Diagnostic errors—the next frontier for patient safety. JAMA. (2009) 301:1060–2. doi: 10.1001/jama.2009.249, PMID: 19278949 PMC12396158

[33] Newman-Toker DE. A unified conceptual model for diagnostic errors: underdiagnosis, overdiagnosis, and misdiagnosis. Diagnosi. (2014) 1:43–8. doi: 10.1515/dx-2013-0027, PMID: 28367397 PMC5373075

[34] Olson APJ Graber ML Singh H. Tracking Progress in improving diagnosis: a framework for defining undesirable diagnostic events. J Gen Intern Med. (2018) 33:1187–91. doi: 10.1007/s11606-018-4304-2, PMID: 29380218 PMC6025685

